# Alleviation of Neuronal Cell Death and Memory Deficit with Chungkookjang Made with *Bacillus amyloliquefaciens* and *Bacillus subtilis* Potentially through Promoting Gut–Brain Axis in Artery-Occluded Gerbils

**DOI:** 10.3390/foods10112697

**Published:** 2021-11-04

**Authors:** Ting Zhang, Myeong-Seon Ryu, Xuangao Wu, Hee-Jong Yang, Su Ji Jeong, Ji-Won Seo, Do-Yeon Jeong, Sunmin Park

**Affiliations:** 1 Department of Bio-Convergence System, Hoseo University, Asan 31499, Korea; zhangting92925@gmail.com (T.Z.); niyani0@naver.com (X.W.); 2 Department of R & D, Microbial Institute for Fermentation Industry, Sunchang 56048, Korea; rms6223@naver.com (M.-S.R.); godfiltss@naver.com (H.-J.Y.); yo217@naver.com (S.J.J.); wldnjs8769@naver.com (J.-W.S.); 3 Department of Food and Nutrition, Obesity/Diabetes Research Center, Hoseo University, Asan 31499, Korea

**Keywords:** fermented soybeans, post-stroke hyperglycemia, inflammation, blood flow, gut microbiota

## Abstract

Short-term fermented soybeans (chungkookjang) with specific *Bacillus (B.)* spp. have anti-obesity, antidiabetic, and anti-stroke functions. We examined the hypothesis that the long-term consumption of *B. amyloliquefaciens* SCGB 1 fermented (CKJ1) and *B. subtilis* SCDB 291 (CKJ291) chungkookjang can alleviate clinical symptoms and hyperglycemia after ischemic stroke by promoting the gut microbiota–brain axis. We examined this hypothesis in Mongolian male gerbils with stroke symptoms induced by carotid artery occlusion. The artery-occluded gerbils were divided into five groups: no supplementation (Control, Normal-control), 4% cooked soybeans (CSB), CKJ1, or CKJ291 in a high-fat diet for 3 weeks. The carotid arteries of gerbils in the Control, CSB, CKJ1, and CKJ291 groups were occluded for 8 min and they then continued on their assigned diets for an additional 3 weeks. Normal-control gerbils had no artery occlusion. The diets in all groups contained an identical macronutrient composition using starch, casein, soybean oil, and dietary fiber. The CSB, CKJ1, and CKJ291 groups exhibited less neuronal cell death than the Control group, while the CKJ1 group produced the most significant reduction among all groups, as much as 85% of the Normal-control group. CKJ1 and CKJ291 increased the blood flow and removal of blood clots, as determined by Doppler, more than the Control. They also showed more improvement in neurological disorders from ischemic stroke. Their improvement showed a similar tendency as neuronal cell death. CKJ1 treatment improved memory impairment, measured with Y maze and passive avoidance tests, similar to the Normal-control. The gerbils in the Control group had post-stroke hyperglycemia due to decreased insulin sensitivity and β-cell function and mass; the CKJ291, CSB, and CKJ1 treatments protected against glucose disturbance after artery occlusion and were similar to the Normal-control. CKJ1 and CKJ291 also reduced serum tumor necrosis factor-α concentrations and hippocampal interleukin-1β expression levels, compared to the Control. CKJ1 and CKJ291 increased the contents of *Lactobacillus*, *Bacillus*, and *Akkermansia* in the cecum feces, similar to the Normal-control. Picrust2 analysis showed that CKJ1 and CKJ291 increased the propionate and butyrate metabolism and the starch and glucose metabolism but reduced the lipopolysaccharide biosynthesis and fatty acid metabolism compared to the Control. In conclusion, daily CKJ1 and CKJ291 intake prevented neuronal cell death and memory dysfunction from the artery occlusion by increasing blood flow and β-cell survival and reducing post-stroke-hyperglycemia through modulating the gut microbiome composition and metabolites to influence the host metabolism, especially inflammation and insulin resistance, protecting against neuronal cell death and brain dysfunction. CKJ1 had better effects than CKJ291.

## 1. Introduction

Stroke is the fourth-largest cause of mortality, and, in the United States in 2018–2019, its primary types were ischemic infarction (87%), primary hemorrhage (10%), and subarachnoid hemorrhage (3%) [1]. On the other hand, Asians have a higher incidence of primary hemorrhage (25%) than Caucasians (10–17%), but the incidence of ischemic infarction increased in different countries of Asia (1.7–16%) during the period 2014–2017 [2]. Stroke incidence increases with age, and after 55 years, the incidence doubles with each subsequent decade; it is higher in men than women [1]. The increase is related to hyperglycemia, dyslipidemia, hypertension, and platelet aggregation with age, and their management prevents ischemic infarction. Ischemic infarction reduces the blood flow, resulting in a decrease in oxygen and nutrients, and it is treated by removing the clot [2]. The treatment delay develops neuronal cell death to induce adverse outcomes, such as vision problems, paralysis in the limbs, dizziness, confusion, and coordination loss due to blockage in the brain areas. Furthermore, post-stroke hyperglycemia exacerbates the post-stroke outcomes by aggravating neuronal cell death through promoting procoagulant platelet formation and reducing cerebral blood flow [3,4]. Therefore, hyperglycemia, dyslipidemia, and hypertension need to be controlled to reduce stroke events and alleviate post-stroke consequences. 

Traditionally made chungkookjang is fermented cooked soybeans with rice straw and no added salts for 2–3 days, while bacteria naturally come from the environment. The predominant bacteria in traditional chungkookjang are *Bacillus* (*B.*) *subtilis*, *B. amyloliquefaciens*, and *B. licheniformis* [5]. Compared to unfermented cooked soybeans, traditionally made chungkookjang is rich in isoflavone aglycones, poly-γ-glutamic acid (γ-PGA), and peptides compared to unfermented cooked soybeans [5,6]. Some *Bacillus* spp. in chungkookjang highly synthesize γ-PGA and are edible, biodegradable, non-toxic, and non-immunogenic [7,8]. γ-PGA has been reported to promote immune response and hypoglycemic agents, and it potentially acts as a prebiotic [7,8]. Chungkookjang with high γ-PGA improves brain insulin resistance and neuroinflammation through the gut microbiome–brain axis [7,8]. Furthermore, some traditionally made chungkookjang with high γ-PGA have various metabolic functions, including reducing hyperglycemia, dyslipidemia, neuronal cell death, and obesity in humans and animals [9,10,11]. However, other traditionally made chungkookjang do not have beneficial functions, possibly due to the less efficient *Bacillus* spp. from the environment. A single *Bacillus* spp. has been examined to produce *Bacillus* inoculated chungkookjang with better functions [12]. Different species and strains of *Bacillus* in chungkookjang have different characteristics and functionalities and have no adverse outcomes [13,14]. Chungkookjang is comparable to Japanese natto, soybeans fermented with *B. subtilis* natto for approximately two days. During the fermentation of soybeans, *B. subtilis* natto produces nattokinase and γ-PGA, which help prevent or treat cardiovascular diseases by improving hypertension and dyslipidemia and reducing blood clotting [15]. *B. subtilis*, *B. licheniformis*, and *B. amyloliquefaciens* also have fibrinolytic activities and produce γ-PGA, but different strains have different efficiencies [7,8]. The optimal *Bacillus* species and strain for preventing blood clotting and alleviating post-stroke outcomes need to be determined for chungkookjang production. 

In previous studies, chungkookjang, fermented soybeans using *B. amyloliquefaciens* SRCM 730 and SRCM 731, contains high γ-PGA, and its intake exhibits antidiabetic activity, anti-ischemic stroke, and anti-Alzheimer’s disease activity in animals [8,16,17,18]. Their intake decreases Enterobacteriales and elevates Bacillales, Lactobacillales, and Verrucomicrobiales (*Akkermansia muciniphila*) in the cecum of type 2 diabetic and Alzheimer’s disease animal models [19]. Improvement of insulin sensitivity and inflammation is modulated with the brain–liver–gut microbiota axis [11]. On the other hand, chungkookjang fermented with different strains of *B. subtilis* have not been studied for glucose metabolism and gut microbiota. The present study tested the hypothesis that the long-term consumption of chungkookjang inoculated with *B. subtilis* SCDB 291 and *B. amyloliquefaciens* SCGB 1 alleviated the ischemic stroke-induced clinical symptoms and post-stroke hyperglycemia through modifying the gut microbiota–brain axis. This hypothesis was examined in gerbils with an induced artery occlusion. 

## 2. Materials and Methods

### 2.1. Cultivation of B. subtilus SCDB 291 and B. amyloliquefaciens SCGB 1 and Their Probiotic Properties 

*B. subtilus* SCDB 291 and *B.* *amyloliquefaciens* SCGB 1 were isolated from traditionally made chungkookjang at the Institute of Sunchang Fermented Soybean Products (Sunchang, Korea). They were registered as KCCM11966 and KCCM 11966P in the Korean Collection for Type Cultures [13,14]. Both *Bacillus* spp. were cultivated in Luria-Bertani (LB) broth (Difco, Sparks, MD, USA) at 37 °C in a shaking bath (128 rpm, Jeio Tech, Daejeon, Korea), and their supernatants were collected after centrifuging the LB media in a high-speed centrifuge (Hanil Science Co., Daejeon, Korea) at 15,000× *g* for 10 min. Because *Bacillus* spp. excrete the enzymes when they are expressed, extracellular enzyme activity (protease, amylase, and cellulase) was measured with the culture media using the agar well diffusion method. The plates were composed with LB-agar media containing 2% skimmed milk (Difco, Sparks, MD, USA) for protease activity, 1% carboxymethyl cellulose (Junsei Chemical Co. Ltd., Chuoku, Japan) for cellulase activity, or 1% soluble starch (Junsei Chemical Co. Ltd., Chuoku, Japan) for amylase activity. The supernatant of the culture media was dotted into the middle of the LB-agar plate containing a specific substrate. The diameters (mm) of the clear zones were measured to determine the enzyme activity [13]. The thrombolytic activity was measured in the supernatants using the agar well diffusion method. The isolated supernatants were dotted into the middle of a 0.5% agarose plate containing thrombin and fibrinogen solution at pH 7.4. The diameters (mm) of the clear zones indicated the thrombolytic activity [15]. The positive-control activity was measured with plasmin. 

The pH tolerance was tested in pH 2.0 with HCl, and bile salt tolerance was assessed with 0.5% oxgall (Difco, Sparks, MD, USA) in the LB medium for 24 h incubation [20]. Each *Bacillus* spp. in LB media was heated from 30 °C up to 80 °C in a water bath. The cell survival percentage was calculated from the values of optical density at 600 nm in a spectrophotometer. Antioxidant activity was estimated from the ability to remove reactive oxygen species (ROS) using 2,2-diphenyl-1-picryl-hydrazyl (DPPH, Sigma-Aldrich, St. Louise, MO, USA), as previously described [20]. 

Adhesion of *Bacillus* spp. to colon cells was conducted with the previously described method [21]. Briefly, human colon-derived CCD-18Co cells were plated into 24-well plates at 2 × 10^5^ cells/well and incubated for 24 h. *Bacillus* spp. were inoculated to the plate at 10:1 multiplicity of infection (MOI), and it was incubated at 37 °C in a 5% CO_2_ atmosphere for 45 min. The wells were washed three times with fresh minimum essential media (MEM) to remove unbound bacteria in the LB media by aspirating the LB media. The number of bacteria was counted under a microscope, and the percentage of the remaining bacteria was calculated [21]. 

### 2.2. Chungkookjang Preparation and Isoflavonoid Contents

*Glycine max* L. (yellow soybeans, Daewon; Sunchang, Korea) was soaked in water (2:5; *w*/*w*) at room temperature for 12 h, sterilized at 121 °C for one hour, and cooled to 37 °C. The 1% (*v*/*w*) *B. subtilis* SCDB 291 or *B.* *amyloliquefaciens* SCGB 1 were inoculated into the cooked soybeans at 10^7^–10^8^ colony-forming unit (CFU)∙mL^−1^ concentrations, and the inoculated cooked soybeans were incubated at 37 °C for 48 h, according to the previous studies [8,22,23]. The fermented soybeans were freeze-dried in a freezing dryer (Il Shin, Daejeon, Korea). Lyophilized cooked soybean or chungkookjang fermented with *B.* *amyloliquefaciens* SCGB 1 or *B. subtilis* SCDB 291 were dissolved in 70% methanol containing 0.1% acetic acid and filtered. Isoflavonoid glycones and aglycones of the supernatants were measured using liquid chromatography-electrospray tandem mass spectrometry (LC-MS/MS) equipped with SM-C18 column (100 × 2 mm, 3 μm). The measurement conditions have been described in the previous study [17]. The standards for isoflavonoids (≥98% purity; Sigma Co., St. Louis, MO, USA) were used, and their contents were quantitated with a validated linear curve. 

### 2.3. Animals and Diets

Fifty male Mongolian gerbils (*Meriones unguiculatus*) aged 7 weeks were purchased from DaehanBio (Eumsung, Korea). They were acclimated to the animal facility for one week at 23 °C, 60% humidity, and a 12-h light/dark cycle. The animals were fed food and water ad libitum. The study was conducted according to the National Institute of Health guidelines, and the Hoseo University Animal Care and Use Committee (HSIAUC-18-065) approved the study protocol. Each group included 10 gerbils. 

The ischemic stroke-induced gerbils were divided randomly into the following groups: (1) artery occlusion + a high-fat diet (Control; *n* = 10), (2) artery occlusion + a high-fat diet supplementing 4% cooked soybeans (CSB; *n* = 10), (3) artery occlusion + a high-fat diet supplementing 4% chungkookjang SCGB 1 (CKJ1; *n* = 10), and (4) artery occlusion + a high-fat diet supplementing 4% chungkookjang SCDB 291 (CKJ291; *n* = 10). Gerbils with sham-operation of artery occlusion were given a high-fat diet as the Normal-control group (*n* = 10). 

Chungkookjang fermented *B. subtilus* SCDB 291 or *B. amyloliquefaciens* SCGB 1, or cooked soybeans (4.0%) were added to make a 43 En% fat diet made with modified semi-purified American Institute of Nutrition (AIN)-93 diets. The macronutrients in chungkookjang and cooked soybeans were tailored to include identical contents of fats, carbohydrates, protein, and dietary fibers by adding starch, casein, soybean oil, and cellulose instead of cooked soybeans or chungkookjang using their nutrient composition. The diets consisted of fats (43 En%), carbohydrates (40 En%), protein (17 En%), cellulose (3.4%), mineral (3.5%), and vitamin (1.0%). Their nutrient composition is provided in Supplemental Table S1.

### 2.4. Transient Forebrain Ischemia Induction

Mongolian gerbils are susceptible to epileptic seizures and have low hippocampal neuronal density and only two common carotid arteries. They are a suitable animal model for studying cognitive impairment and neuronal cell death by occluding two carotid arteries to induce ischemic stroke [24,25]. Anesthesia with chloral hydrate does not affect body temperature during artery occlusion, to protect against neuronal cell death [26,27]. The gerbils were given an intraperitoneal injection of 400 mg/kg bw chloral hydrate (Sigma, St. Louis, MO, USA), and they had an occlusion of two carotid arteries for eight min. After making a midline incision of the neck skin, transient ischemic stroke was induced when the common carotid arteries of the gerbils were occluded with aneurysm clips for 8 min [26,27]. The rectal temperature was monitored to maintain 37 ± 0.5 °C with a rectal temperature probe (TR-100; Fine Science Tools, Foster City, CA, USA) during the artery occlusion [26]. After artery occlusion, the gerbils were placed in a thermal incubator (Mirae Medical Industry, Seoul, Korea) to maintain their body temperature for 12 h. Sham surgery was performed with no occlusion of the common carotid arteries. 

### 2.5. Experimental Design, Glucose Metabolism, Memory Impairment, and Grip Strength 

**Figure 1:**
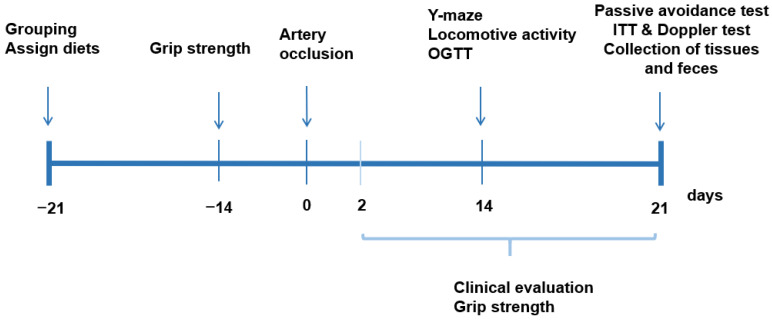
Experimental design.

Figure 1 presents the experimental design. All gerbils had 21 days of the assigned diets. At 22 days, they had the artery occlusion or its sham occlusion, and they consumed the same assigned diet for an additional 21 days. The experimental day was counted as the reference of artery occlusion (Figure 1). Body weight, food intake, and overnight-fasted serum glucose levels were measured weekly. Clinical neurological symptoms and grip strength were evaluated at 2, 7, 14, and 21 days after the artery occlusion. Y maze, passive avoidance tests, locomotive activity, and oral glucose tolerance tests (OGTT) were performed on the assigned days (Figure 1). On the 17th day of the artery occlusion, overnight-fasted gerbils were orally fed 2 g glucose/kg body weight, and serum glucose and insulin concentrations were measured, as previously described [28]. The gerbils were freely fed food and water after the OGTT was finished. On the following day of OGTT, the gerbils had no food for a 6 h, and 1 IU/L insulin was intraperitoneally injected, and the serum glucose concentrations were measured every 15 min; this is called an intraperitoneal insulin tolerance test (IPITT), as described previously [28]. Serum glucose levels were measured using a Glucose Analyzer II (Beckman, Palo Alto, CA, USA), and serum insulin concentrations were determined with an ultrasensitive rat and mouse insulin kit (Crystal Chem, Elk Grove Village, IL, USA). 

### 2.6. Neurological Severity Score and Grip Strength

The neurological severity was clinically evaluated: eyelid droop (0, no symptom; 1, one eyelid partially drooped; 2, one eyelid completely drooped; 3, both eyelids partially drooped; 4, both eyelids completely drooped); hair bristling (0, no symptom; 1, hair bristled); decreased muscular tone (0, no symptom; 1, reduced muscular tone or strength in the limbs); flexor reflex (0, no symptom; 1, slight withdrawal of hind limbs when pinched; 2, no withdrawal of hind limbs when pinched); posture (0, normal; 1, hunched); walking pattern (0, normal; 1, slow; 2, not moving). 

At 2, 14, and 21 days after artery occlusion, a researcher slowly pulled the tail of a gerbil when the gerbil forelimbs grasped the bar mounted on a force gauge of a Grip Strength Meter (GPM-100; Melquest, Toyama, Japan). Peak pull force by forelimb was measured in grams to represent the grip strength of the forelimb [29]. 

### 2.7. Short-Term Memory Impairment Measurement Using the Y maze and Passive Avoidance Tests

On the 14th day, the Y maze test was assessed in a horizontal Y-shaped maze with three arms composed of 20 cm in width, 50.5 cm in length, and 20 cm in height. A gerbil was located in one arm, and the movements in each arm were monitored for 8 min. The number of correct consecutive entries into each arm was measured, and the percentage of the right consecutive alternation in total alternations was calculated [30]. Its higher percentage indicated a better short-term memory. 

On the 19th day, the passive avoidance test was carried out in an apparatus composed of a dark and light compartment shuttle box [31]. When entering the dark chamber in the first trial, a gerbil was given an electric shock having 0.2 mA, 75 V, and 50 Hz for five seconds. After 8 h later, the second trial was conducted the same as the first trial. After 16 h from the 2nd trial, the latency time entering the dark compartment was similarly measured to the 1st trial without electric shock. Latency periods to the darkroom were determined up to 600 s. 

### 2.8. Lipid Peroxidation Contents 

Malondialdehyde (MDA) contents of the hippocampus were measured. MDA formed a colored complex with TBA, and the color was measured at 532 nm in a spectrophotometer (Perkin Elmer, Waltham, MA, USA). The hippocampal MDA contents were given as μmol/g protein in the hippocampus using 1,1′,3,3′-tetraethoxypropane as a standard.

### 2.9. Cresyl Violet Staining in the Hippocampus

Neuronal cell death was evaluated with cresyl violet in the hippocampal sections at the end of the total 42-day experimental period. The brains were soaked overnight in a 30% sucrose solution and then frozen. The frozen brains had a serial section of 30-µm on a cryostat (Leica, Wetzlar, Germany), and the randomly selected sections were mounted on gelatin-coated microscopy slides [31]. After staining the sections with a 0.1% cresyl violet solution in 0.6% acetic acid for 2 min at room temperature, they were washed twice in distilled water. The fixed tissues were dehydrated with a graded series of ethanol at room temperature and mounted with cover glass with Permount (Fisher Scientific Inc., Pittsburgh, PA, USA).

### 2.10. Islet Morphometry by Immunohistochemistry 

Four gerbils randomly selected from each group, were injected with 100 µg/kg body weight 5-bromo-2-deoxyuridine (BrdU; Roche Molecular Biochemicals) at the end of the experimental period. Six hours post-injection, the dissected pancreas was fixed in a 4% paraformaldehyde solution (pH 7.2) at room temperature for 16 h and embedded in paraffin blocks. Serial 5-μm paraffin-embedded tissue sections were mounted on slides, and they were rehydrated for the immunohistochemistry method. 

Every sixth or seventh pancreas section was incubated with a guinea pig anti-insulin antibody. The anti-insulin antibody-stained areas of the pancreas were measured using anti-insulin (Zymed Laboratories, South San Francisco, CA, USA), and the percentage of the stained area from the total pancreas area was calculated. The β-cell proliferation was identified by incorporating BrdU into β-cells using the anti-BrdU antibody (Roche, Mannheim, Germany). The β-cell apoptosis was determined using a TUNEL assay (Roche) and double-stained with hematoxylin and eosin to visualize the islets [17]. The BrdU^+^ incorporated cells and apoptotic bodies of the islets were quantified based on the total islet numbers [27]. 

### 2.11. Serum Short-Chain Fatty Acid (SCFA) Concentrations by Gas Chromatography (GC)

Serum concentrations of acetate, propionate, and butyrate contents were measured by GC (Clarus 680 GAS, PerkinElmer) using an Elite-FFAP capillary column with 30 m × 0.25 mm × 0.25 μm, with helium as the carrier gas at a flow rate of 1 mL/min, as described elsewhere [17]. The supernatants containing SCFA were separated from the serum with a solvent containing acetonitrile: n-butanol: tetrahydrofuran (20:50:30; Waters, Milford, MA, USA) in acid solution. 

### 2.12. Next-Generation Sequencing (NGS) of gut Microbiomes 

The gut microbiome composition of the cecum feces was measured using NGS, as described elsewhere [17,28]. Fecal bacteria were identified and counted from NGS results using the Miseq standard operating protocol described in the previous study [28]. Mothur v.1.36 was used to analyze 16S amplicon sequencing [28]. The sequences were aligned using Silva reference alignment v.12350, and the operational taxonomic units (OTUs) were selected with the criteria of 98% identity and taxonomically classified by consensus using the Silva reference. Principal Coordinates Analysis (PCoA) was performed using the R package with the OTU-abundance table converted to the relative abundance. 

### 2.13. Statistical Analysis 

Statistical analysis was conducted with SAS software (Cary, NC, USA). All results were represented as the means ± standard deviation (SD). The significance of the treatment effects on the clinical symptoms was performed by one-way ANOVA followed by multiple comparisons with a Tukey’s test. The statistical differences among the groups were significant at the level of a *p* < 0.05. 

## 3. Results

### 3.1. Characteristics of B. amyloliquefaciens SCGB 1 and B. Subtilis SCDB 291 

The protease and cellulase activities were similar in *B. amyloliquefaciens* SCGB 1 and *B. subtilis* SCDB 291 (Table 1). On the other hand, *B. subtilis* SCDB 291 has higher thrombolytic and antioxidant activities than *B. amyloliquefaciens* SCGB 1. The percentages of surviving cells at 0.5% oxygal, low acidity (at pH 2), and hot temperature (80 °C) were high, but those were much higher in *B. subtilis* SCDB 291 than in *B. amyloliquefaciens* SCGB 1 (Table 1). Both *Bacillus* spp. showed no bile salt hydrolase activity. Cell adhesion to CCD-18Co was 62.1 and 82.1% in *B. amyloliquefaciens* SCGB 1 and *B. subtilis* SCDB 291, respectively. Both *Bacilli* did not express *B. cereus*-related genes. γ-PGA contents were much higher in *B. amyloliquefaciens* SCGB 1 than in *B. subtilis* SCDB 291 (Table 1). Therefore, *B. amyloliquefaciens* SCGB 1 and *B. subtilis* SCDB 291 met the probiotics criteria, although *B. subtilis* SCDB 291 exhibited better probiotic characteristics than *B. amyloliquefaciens* SCGB 1. However, γ-PGA contents were higher in *B. amyloliquefaciens* SCGB 1. 

### 3.2. Energy Metabolism 

The body weight gain was not different between the Control and Normal-control groups but was much higher in the CSB, CKJ1, and CKJ291 groups than in the Control during the first three weeks before artery occlusion (Table 2). After artery occlusion, body weight gain was lower in the Control than in the Normal-control, whereas CKJ1 and CKJ291 prevented the decrease. The food intake was higher in the CKJ1 and CKJ291 than the other groups before artery occlusion, while food intake was similar after artery occlusion (Table 2). CKJ1, CKJ291, and CSB were approximately 2.5 g/kg body weight/day, equivalent to approximately 20 g/day for humans. CSB, CKJ1, and CKJ291 consumed isoflavonoids (Table 1). Isoflavonoid aglycone intake was much higher in CKJ1 and CKJ291 than in CSB, while isoflavonoid glycone intake was opposite to isoflavonoid aglycone intake. 

### 3.3. Neuronal Cell Death in the Hippocampus and Memory Dysfunction

**Figure 2:**
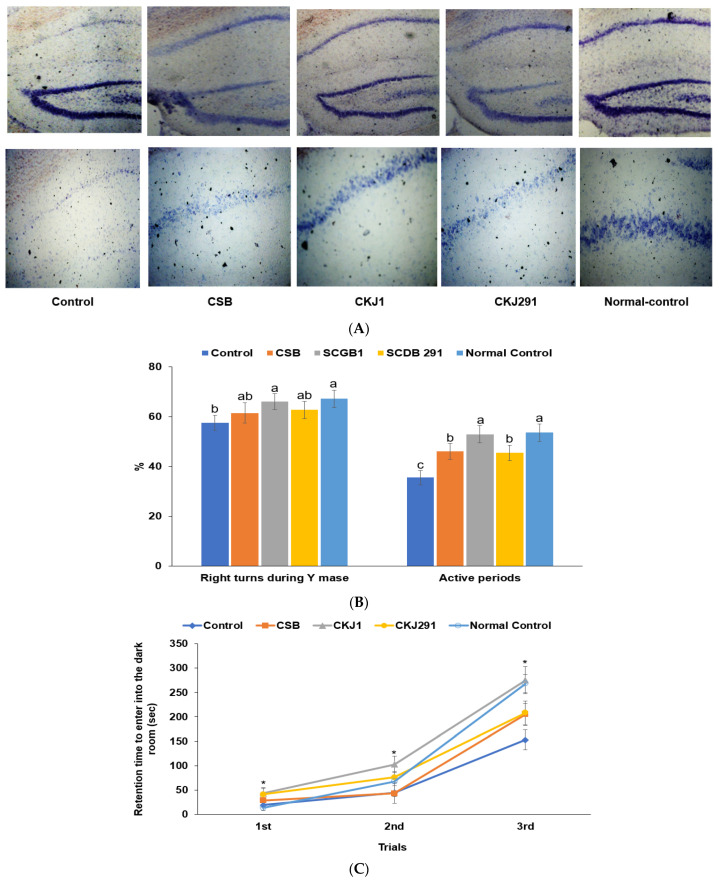
Brain cell death after ischemic stroke and memory loss. On the 3rd week after inducing artery occlusion, the survived cells were quantified in a cresyl violet stained brain section by densitometry (**A**). The spontaneous alternations in the Y maze and active periods in locomotive activity were measured on the 14th and 15th day after artery occlusion (**B**). The passive avoidance tests were conducted three times (**C**). Dots and bars represent means ± SD (*n* = 10). * Significantly different among the groups at *p* < 0.05. ^a,b,c^ Means on the bars with different letters were significantly different by Tukey’s test at *p* < 0.05.

The hippocampal live neuronal cells showing blue staining increased in the ascending order of the Control, CSB, CKJ291, CKJ1, and Normal-control (*p* < 0.05; Figure 2A). As determined by the Y maze (Figure 2B) on the 14th day and passive avoidance tests on the 19th day after artery occlusion (Figure 2C), the artery-occluded gerbils exhibited short-term memory disorder. The correct alteration among total alteration (%) during the Y maze test was much lower in the Control than in the Normal-control, whereas the CKJ1 group showed an improved percentage of the correct alteration similar to the Normal-Control (*p* < 0.05; Figure 2B). The active time during the locomotive activity was lowest in the Control because they were lethargic from the ischemic stroke. The active movement period was longer in the CSB and CKJ291 groups than in the Control group, while it was longest among the groups (Figure 2B). The latency entering the darkroom was shorter in the Control than in the Normal-Control, while it protected against its decrement in CSB, CKJ291, and CKJ1, similar to the Normal-control (Figure 2C). These results suggested that CKJ1 and CKJ291 treatment alleviated the short-term memory impairment in artery-occluded gerbils, and CKJ1 protected against memory dysfunction as much as in the Normal-control. 

### 3.4. Clinical Neurological Symptom Evaluation 

**Figure 3:**
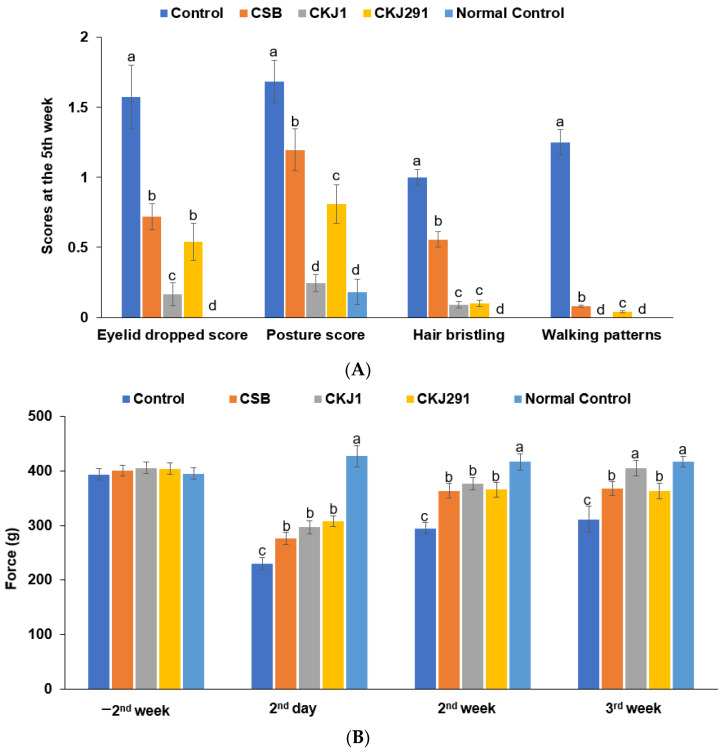
Neurological severity scores. The neurological symptoms, including drooping eyelids, crouched posture, bristling hair, flexor reflex and walking patterns (**A**), and force to grip the bar (**B**), were shown on the 2nd day, 2nd week, and 3rd week after inducing an ischemic stroke. Bars represent means ± SD (*n* = 10). ^a,b,c,d^ Means on the bars with different letters were significantly different by Tukey’s test at *p* < 0.05.

Eyelid drooping, crouched posture, hair bristling, and slow walking were observed in the artery-occluded gerbils. All gerbils showed improved symptoms every week, but the improvement rate was different between the groups. The scores of the clinical neurological symptoms at the 3rd week after artery occlusion were much higher in the Control than in the Normal-control. CSB, CKJ1, and CKJ291 reduced the scores of the clinical neurological symptoms, including drooping eyelids, crouched posture, hair bristling, and slow walking patterns (Figure 3A). However, the improvement by CKJ1 and CKJ291 did not reach the Normal-control (Figure 3A). CKJ1 and CKJ291 exhibited lower scores than CSB and CKJ1 improved the symptoms better than CKJ291 (*p* < 0.05; Figure 3A). 

The grip strength holding the bar of the Control group was approximately two times lower than the Normal-control group at two days after artery occlusion. The grip strength increased in the Control over time. In the 3rd week, the grip strength remained lower in the Control than in the Normal-control. The grip strength elevated more in the CSB, CKJ1, and CKJ291 groups than in the Control, and CKJ1 elevated grip strength up to the Normal-control level in the 3rd week (*p* < 0.05; Figure 3B). 

These neurological differences were involved in brain cell death related to the lipid peroxide amounts and proinflammatory cytokine mRNA expression in the hippocampus. Serum IL-1 concentrations and inflammatory index were higher in the Control than in the Normal-control. CSB, CKJ1, and CKJ291 lowered the serum IL-1 concentrations and inflammatory index as much as in the Normal-control (Table 3). The serum TNF-α concentrations showed a similar trend to the serum IL-1 concentrations. The hippocampal lipid peroxide contents were higher in the Control than in the Normal-control, while they were lower, in descending order, in the Control, CSB, CKJ291, and CKJ1 (Table 3). The relative mRNA expression of IL-1β and TNF-α decreased in the CSB, CKJ291, and CKJ1, while it was similar between CKJ1 and Normal-control (Table 3). 

### 3.5. Blood Flow and Lipid Profiles

Peak blood perfusion unit (BPU), the capability to remove a blood clot, was much lower in the Control group than in the Normal-control group, and CSB, CKJ1, and CKJ271 increased the levels compared to the Control on the 21st day after artery occlusion. BPU in the CKJ271 group was similar to the Normal-control (Table 4). The period to remove the blood clot was longer in the Control than in the Normal-control, while it lowered in CSB, CKJ1, and CKJ291 compared to the Control (Table 4). The period of the CKJ1 and CKJ291 groups was similar to the Normal-control. The lipid profiles were disturbed in the Control compared to the Normal-control, while CSB, CKJ1, and CKJ291 offered protection against dyslipidemia in the Control (Table 4). Interestingly, the serum HDL concentrations were higher in CKJ1 and CKJ291 but not in CSB, whereas the serum triglyceride concentrations were lowest in CSB.

### 3.6. Glucose Metabolism

**Figure 4:**
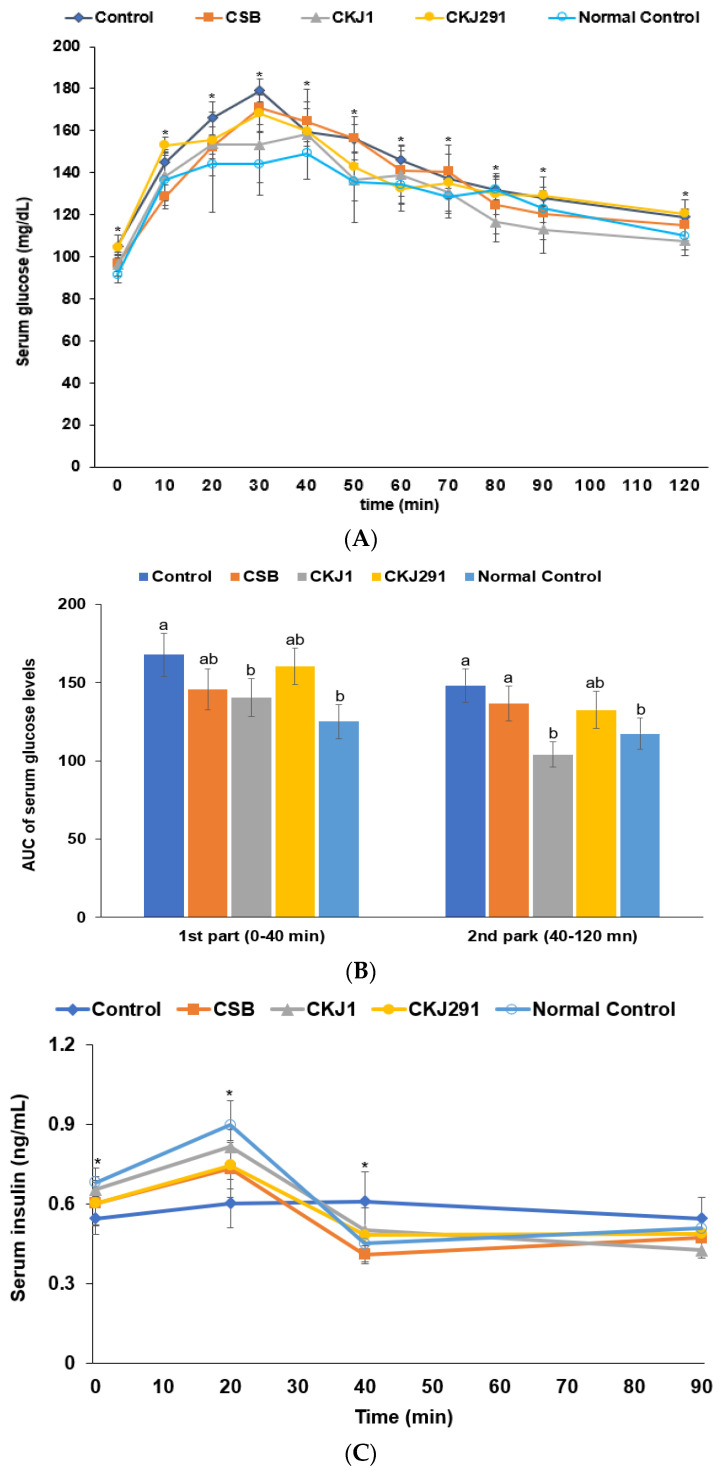

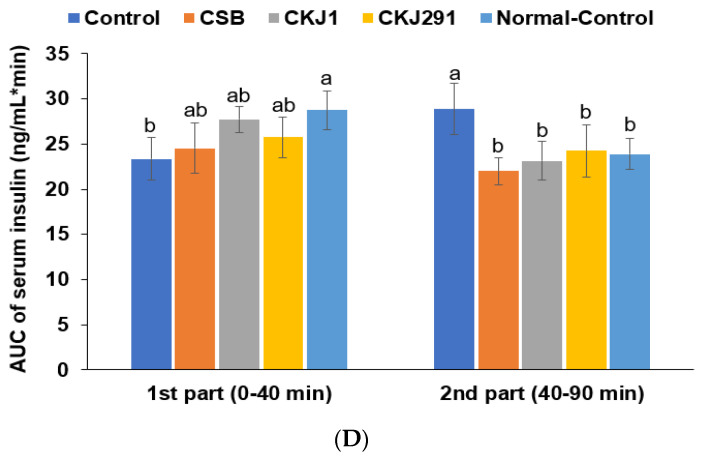
Oral glucose tolerance test (OGTT). Gerbils underwent an OGTT with 2 g glucose/kg body weight after overnight fasting on the 17th day after artery occlusion. The changes of serum glucose levels during 120 min (**A**) and area under the curve (AUC) of serum glucose concentrations in the first part (0–40 min) and the second part (40–120 min) during OGTT (**B**). The changes of serum insulin concentrations during 120 min (**C**) and AUC of serum insulin concentrations (**D**). * Significantly different among the groups at *p* < 0.05. Bars and dots represent means ± SD (*n* = 10). ^a,b^ Means on the bars with different letters were significantly different by Tukey’s test at *p* < 0.05.

On the 3rd weeks before and after artery occlusion, the serum glucose concentrations were lower, in the following order: Control, CKJ291, CSB, CKJ1, and Normal-control. On the other hand, the serum insulin concentrations were lower in the Control than in the Normal-control (Table 4; Figure 4A), whereas the other treatments increased them. CKJ1 increased the serum insulin concentrations to those of the Normal-control. After an oral challenge of 2 g glucose on the 17th day after artery occlusion, the peak of serum glucose concentrations at 30 min was higher in the Control group than in the other treatment groups and decreased with time. The peak was lowest in the Normal-control and CKJ1 (Figure 4A). The decrease was lower in the Control group than in the other groups. CKJ1 decreased the serum glucose concentrations quickly after the peak value, even compared to the Normal-control group (Figure 4A). The AUC of the serum glucose concentrations during the first part of OGTT (0–40 min) was higher in the Control group than in the Normal-control, while CKJ1 reduced the AUC as much as the Normal-control (Figure 4B). The AUC of serum glucose concentrations in the second part was similar to that in the first part (Figure 4B). The serum insulin concentrations during the OGTT increased until 20 min and then decreased until 40 min, which is called the acute phase. The serum insulin concentrations then increased after 40 min until 90 min. The serum insulin concentrations at the peak were higher in the Normal-control than in the Control, and CKJ1 showed similar peak values to the Normal-control (Figure 4C). The AUC of the serum insulin concentrations was lower in the Control group than in the Normal-control, and CKJ1 increased the AUC as much as the Normal-control (Figure 4D). However, the AUC at the 2nd phase was higher in the Control than in the Normal-control, whereas CKJ treatment showed a similar AUC to the Normal-control (Figure 4D). 

**Figure 5:**
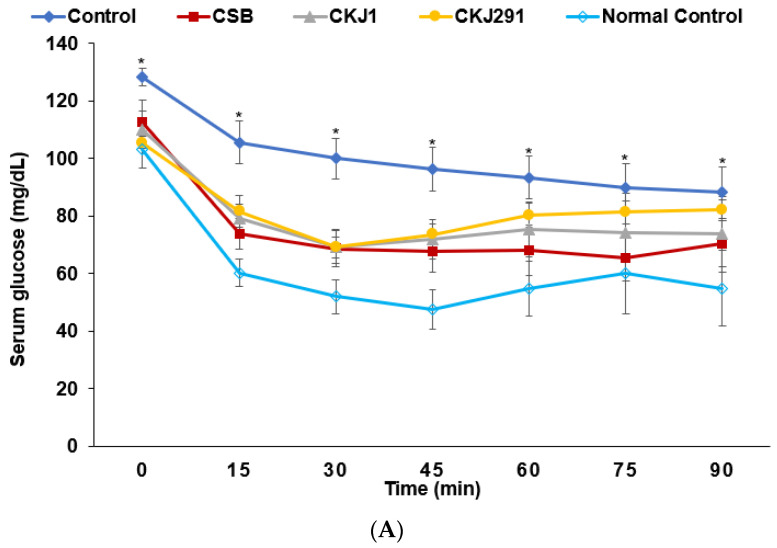

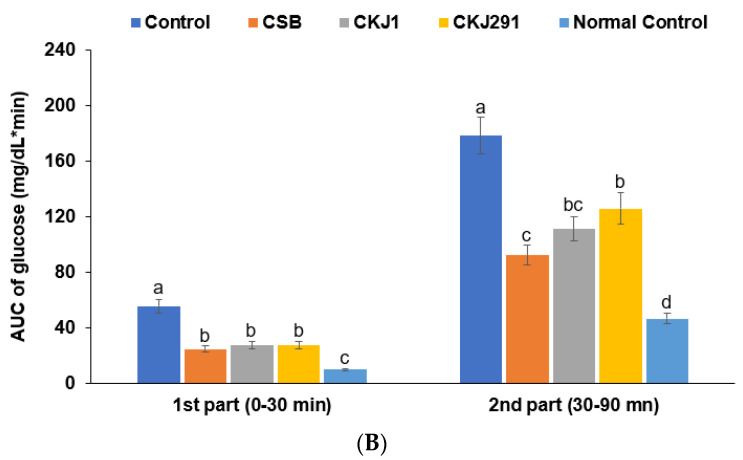
Intraperitoneal insulin tolerance test (IPITT). The gerbils underwent an IPITT with 1 IU insulin/kg body weight after 6 h food deprivation on the following day of OGTT. The changes of serum glucose levels during 90 min (**A**) and AUC of serum glucose in the first part (0–30 min) and the second part (30–90 min) during the IPITT (**B**). * Significantly different among the groups at *p* < 0.05. Bars and dots represent means ± SD (*n* = 10). ^a,b,c,d^ Means on the bars with different letters were significantly different by Tukey’s test at *p* < 0.05.

The insulin sensitivity was determined using the intraperitoneal insulin tolerance test (IPITT) after 6 h of food deprivation on the 19th day after artery occlusion. The serum glucose concentrations were higher in the Control group than in the other groups at the baseline. After injecting insulin intraperitoneally, the serum glucose levels were markedly lower until 30 min and were then maintained or rebounded in all the gerbils examined (Figure 5A). The serum glucose levels at 0, 15, 60, and 90 min were significantly higher in the Control group than in the other groups (Figure 5A). The serum glucose concentrations at 15, 60, and 90 min were lower in the CSB, CSK1, and CKJ291 groups than in the Normal-control group (Figure 5A). The 1st part AUC of the serum glucose concentrations was lower, in the following order: Control, CSB, CKS1, CKJ291, and Normal-control. The second phase of the AUC was also highest in the Control, but was lower in the order of CKJ, CKJ291, CSB, and Normal-control (*p* < 0.05; Figure 5B). 

### 3.7. β-Cell Mass

The individual β-cell size was larger in the Control than in the Normal-control, and CSB and CKJ1 suppressed the increase in individual β-cell size (Table 5). On the other hand, the total β-cell area, which was determined by the individual cell size and the number of β-cells, was lower in the Control than in the Normal-control and was higher in CSB, CKJ1, and CKJ291, as much as in the Normal-control (*p* < 0.05; Table 5). The total β-cell mass, measured by multiplying the total β-cell area by the pancreas weight, was lower in the Control than in the Normal-control, while the decrease was prevented with CKJ1, CSB, and CKJ291 (*p* < 0.05; Table 5). The net β-cell proliferation and apoptosis determine the pancreatic β-cell mass. CKJ1 protected against the lowered β-cell proliferation in the Control, similar to the Normal-control. β-cell apoptosis was higher in the Control than in the Normal-control, whereas CSB and CKJ1 prevented this increase as much as in the Normal-control (*p* < 0.05; Table 5). 

### 3.8. Serum SCFA Concentrations and Gut Microbiome 

Serum acetate and propionate concentrations were not significantly different between the groups (Table 6). However, serum butyrate concentrations were higher in CSB and CKJ1 than in the other groups (Table 6). 

**Figure 6:**
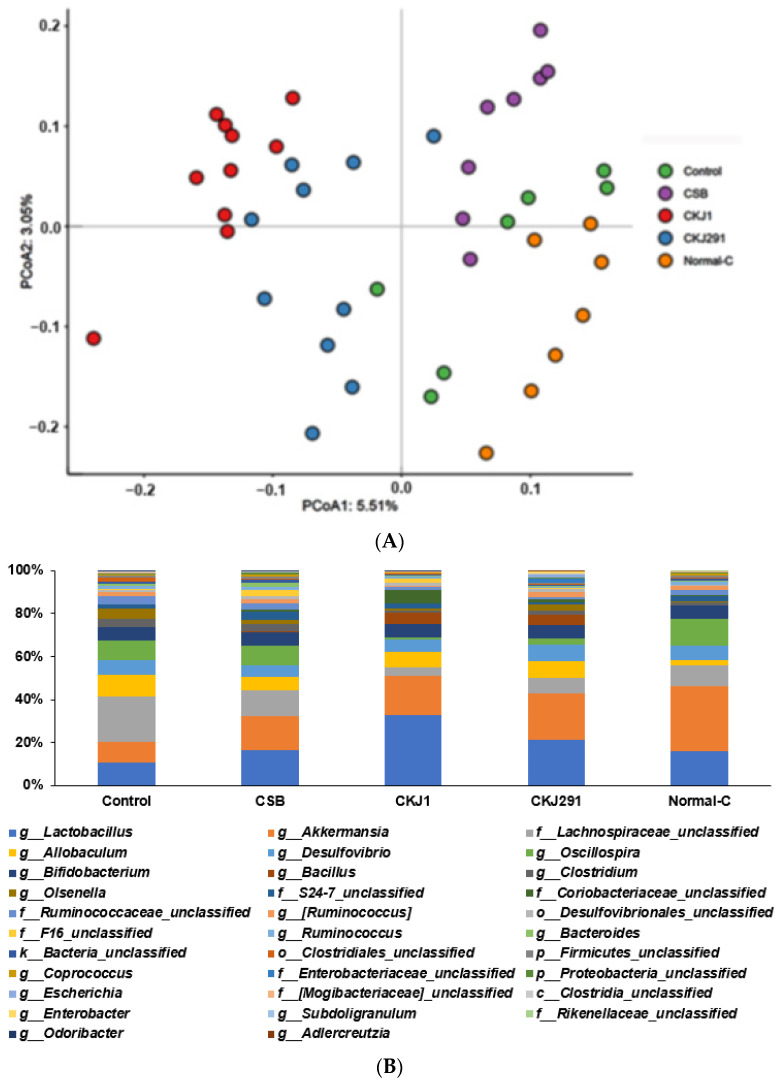
The profiles of gut microbiomes. The principal coordinate analysis (PCoA) of the fecal bacterial community (**A**) and the relative abundance of gut bacteria at the genus level (**B**) were analyzed from the feces in the cecum. Bars represent means (*n* = 10).

The α-diversity of the gut microbiome, the Shannon and Chao index, was lower in the Control than in the Normal-control. CSB, CKJ1, and CKJ291 increased the α-diversity indices compared to the Control (Table 6). In β-diversity, all groups were separated significantly (Figure 6A). The genus level of bacteria was observed in Figure 6B. The relative abundance of *Lactobacillus* and *Akkermansia* among gut bacteria was much lower in the Control than in the Normal-control, whereas CKJ1 and CKJ291 increased it. On the other hand, the relative abundance of Lachanospiracae and *Allobaculum* was higher in the Control than in the Normal-control, and lower in the CSB, CKJ1, and CKJ291 groups (Table 6). The relative abundance of *Bacillus* was higher in CKJ1 and CKJ291 compared to other groups. These results suggested that *Bacillus* in CKJ1 and CKJ291 were grown in the gut. The relative abundance of *Oscillospira* was lower in CKJ1 and CKJ291 than in the Control and Normal-control (Table 6). 

Metagenome analysis with Picrust2 showed that the butanoate metabolism was significantly lower in the Control than in the Normal-control, and higher in the CSB and CKJ1 groups, similar to the Normal-control (Table 6). LPS biosynthesis was higher in the Control than in the other groups. The starch metabolism increased in the order of Control, CSB, Normal-control, CKJ291, and CKJ1, whereas the fatty acid metabolism was lower in the CKJ1 and CKJ291 than in the other groups (Table 6). Branched-chain amino acid synthesis was higher in the Control than in the Normal-control, whereas CSB, CKJ1, and CKJ reduced it. CKJ1 produced the most significant reduction (Table 6). 

## 4. Discussion

Chungkookjang is traditionally fermented with rice straw with various *Bacillus* spp., but the *Bacillus* spp. can be different. The quality of chungkookjang cannot be controlled in traditionally made chungkookjang. The *Bacillus* spp. from various traditionally made chungkookjang have been isolated, and they are used to produce inoculated chungkookjang with one *Bacillus* spp. The primary *Bacillus* in the traditionally made chungkookjang are *B. subtilis*, *B. licheniformis*, and *B. amyloliquefaciens*, but different strains have different functions. Chungkookjang fermented with *B. amyloliquefaciens* SCGB 730 and 731 has antidiabetic and anti-Alzheimer’s disease activity, possibly with the gut–liver–brain axis, by increasing butanoate and decreasing LPS [8,17]. The present study showed that CKJ1 and CKJ291 alleviated the ischemic stroke symptoms and post-stroke hyperglycemia. It was at least partly related to modulating the gut microbiota–brain axis by reducing inflammation and insulin resistance, alleviating intestinal paralysis, and increasing butyrate production. CKJ1 showed better activity than CKJ291. 

A preliminary study isolated *B. amyloliquefaciens* SCGB 1 and *B. subtilis* SCDB 291 from traditionally made chungkookjang. High-γ-PGA containing chungkookjang intake improved glucose regulation and neuronal cell survival in diabetic rats [19]. *B. amyloliquefaciens* SCGB 1 produced γ-PGA at a higher rate than *B. subtilis* SCDB 291 in the present study. Post-stroke hyperglycemia and neuronal cell death were alleviated more in CKJ1 than in CKJ291, which might be associated with their γ-PGA contents. The γ-PGA contents were higher in the CKJ1 than in the CKJ291. *B. subtilis* SCDB 291 had higher fibrinolytic and antioxidant activities than *B. amyloliquefaciens* SCGB 1, whereas *B. subtilis* SCDB 291 was more resistant to bile salt, low acidity, and high temperatures than *B. amyloliquefaciens* SCGB 1. *B. subtilis* SCDB 291 had better adhesion activity than *B. amyloliquefaciens* SCGB 1. Both *Bacilli* met the criteria of probiotics, but *B. subtilis* SCDB 291 acted as a better probiotic than *B. amyloliquefaciens* SCGB 1 [18]. Therefore, both *Bacillus* spp. were used to ferment soybeans to produce chungkookjang. 

CKJ1 and CKJ291 can be used as synbiotics for experimental animals and humans because fermented soybeans contain proteins, dietary fiber, isoflavones, and *Bacillus* spp. In the present study, CKJ1 and CKJ291 acted as synbiotics to improve glucose homeostasis and neuronal cell survival. CKJ1 had better efficacy than CKJ291 in gerbils with an artery occlusion, even though *B. subtilis* SCDB 291 had better antioxidant and fibrinolytic activity. Furthermore, although *B. subtilis* SCDB 291 was more resistant to acid and bile salts and had higher cell adhesion than *B. amyloliquefaciens* SCGB 1, *Bacillus* spp. was higher in the gut of the gerbils in the CKJ1 and CKJ291 groups than in the Control and Normal-control groups. The results suggest that CKJ1 and CKJ291 acted as synbiotics to alter the gut microbiota and improve glucose metabolism and neuronal cell survival. CKJ1 had better activity in alleviating the clinical symptoms of ischemic stroke than CKJ291. A previous study reported that CKJ1 had a similar effect to chungkookjang fermented *B. amyloliquefaciens* SRCM 730 and 731, which positively affected glucose homeostasis and neuronal cell death [8,17]. These results suggest that *B. amyloliquefaciens* may be better for glucose metabolism than *B. subtilis.*

After an artery occlusion, quick normalization of blood flow protects against neuronal cell death by reducing inflammation and oxidative stress in the brain. Blood flow is normalized by increasing BPU and removing the blockage of blood flow. CKJ291 increased BPU more than CKJ1, but the removed blood clots were similar in CKJ1 and CKJ291. On the other hand, CKJ1 exhibited less global neuronal cell death in the brain than CKJ291. The protection was associated with lower lipid peroxides in the brain tissues of the CKJ1 group. Previous studies showed that antioxidants, such as ebselen and N-acetylcysteine, reduced neuronal cell death by cerebral infarction by reducing autophagic activation [27,32]. Neuroinflammation promotes neuronal cell death by infiltrating various inflammatory cells into the ischemic regions after ischemic stroke [33]. The decrease in post-stroke neuroinflammation ameliorates neuronal cell death and clinical symptoms after ischemic stroke. Consistent with neuronal cell death, short-term memory deficits were impaired in the gerbils after artery occlusion, and CKJ1 alleviated the impairment the most, even though CSB, CKJ1, and CKJ291 had beneficial activity in memory impairment. CKJ1 showed better improvement of oxidative stress than CKJ291, but the neuroinflammation measured by hippocampal IL-1β and TNF-α expression was similar in both groups. 

Post-stroke hyperglycemia is related to poor clinical outcomes in experimental animals and patients. Hyperglycemia exacerbates the calcium imbalance and oxidative stress in the brain to increase neuronal death, and it induces lactic acidosis by activating the anaerobic pathway to produce energy [34]. The smaller amount of neuronal cell death in the CKJ1 may be involved in post-stroke hyperglycemia. Post-stroke hyperglycemia, by reducing insulin resistance and improving the β-cell function and β-cell mass, was better in CKJ1 than in CKJ291. Ischemic stroke also induces β-cell apoptosis to reduce the β-cell mass and impair β-cell function [26,35,36]. These changes were associated with post-stroke hyperglycemia. Chungkookjang has been reported to improve insulin sensitivity and potentiate glucose-stimulated insulin secretion in experimental animals and clinical studies [6]. Different species and strains of *Bacillus* have different potencies on glucose metabolism. CKJ1 improved glucose metabolism better than CKJ291, possibly by preventing β-cell apoptosis. 

Chungkookjang intake modulates the gut microbiomes. In an in vitro study, a chungkookjang treatment effectively enhanced the abundance of beneficial bacteria, such as *Coprococcus*, *Ruminococcus*, and *Bifidobacterium*, but inhibited opportunistic pathogens *Sutterella*, *Escherichia/Shigella*, and *Collinsella* [37]. By contrast, the present study showed that CKJ1 and CKJ291 intake modulated gut bacteria in gerbils differently, even though the relative abundance of beneficial bacteria was elevated. CKJ1 and CKJ291 intake increased the relative abundance of beneficial bacteria such as *Lactobacillus*, *Akkermansia*, and *Bacillus* but decreased *Oscillospira*. These changes in gut microbiota were similar to the previous study on the intake of chungkookjang fermented with *B. amyloliquefaciens* SRCM730 and 731 in Alzheimer’s disease-induced rats [19]. CKJ291, but not CKJ1, increased the relative abundance of some proteobacteria, including *Escherichia* and *Enterobacteriaceae*. As *Oscillospira* was reported to cause constipation and a low BMI, CKJ1 and CKJ291 showed consistent body weight results. CKJ1 and CKJ291 may be beneficial for constipation. *Akkermansia* is a mucin-degrading bacterium and releases a glucagon-like peptide-1 producing protein to improve glucose metabolism. The CKJ1 and CKJ291 intake elevated the relative abundance of *Akkermansia* to improve glucose homeostasis [38]. CKJ1 and CKJ291 also increased the relative abundance of *Lactobacillus* spp., a well-known probiotic, to improve glucose metabolism [39]. *Lactobacillus (L.) rhamnosus*, *L. sakei*, and *L. acidophillus* regulate glucose metabolism in diabetic animal models [40,41]. Therefore, CKJ1 and CKJ291 modulated the gut microbiota to improve the glucose metabolism in the present study, and CKJ1 modulated gut microbiota that were more beneficial to the glucose metabolism than CKJ291. 

Artery occlusion induces ischemia directly to increase neuronal cell death by inflammation and oxidative stress in the brain, leading to intestinal paralysis and gut dysbiosis [42]. A high-fat diet aggravates the gut changes [43]. The intestinal paralysis induces gut microbiome dysbiosis, which in turn causes intestinal barrier permeability, allowing endotoxins and trimethylamine to enter the host circulation easily, thereby elevating proinflammatory cytokines, platelet hyperreactivity, and overactive immunity [44]. The changes send signals directly to the brain through the autonomous nervous system and SCFA production by the gut microbiome [45]. This is known as the gut microbiome–brain axis. The changes aggravate neuronal cells and cause β-cell death, which exacerbates ischemic stroke outcomes [45]. Chungkookjang intake may modulate the gut microbiome-brain axis by preventing gut microbiome dysbiosis and possibly intestinal paralysis in the present study. We previously demonstrated that chungkookjang intake improves the intestinal morphology, gut microbiome, glucose tolerance, and β-cell function and survival in diabetic rats, possibly due to its γ-PGA, soluble fiber, *Bacilli*, and isoflavonoids [17]. In the present study, CKJ1 and CKJ271 intake alleviated the clinical outcomes of artery occlusion, which may indirectly promote the gut microbiome–brain axis in an animal model. However, intestinal paralysis was not measured in the present study, although serum SCFA concentrations and beneficial bacteria in the gut microbiome were promoted and associated with reduced insulin resistance and inflammation. However, there is no clearly established cause and effect between the microbiome benefits and the stroke outcomes. Therefore, more research is needed to confirm the activation of the gut microbiome–brain axis. 

## 5. Conclusions

Daily CKJ1 and CKJ291 intakes protect against neuronal cell death by increasing blood flow and β-cell function and reducing post-hyperglycemia. Isoflavonoids and peptides can suppress neuronal cell death directly. The protection was also associated with a change in the gut microbiome. CKJ1 exhibited better protective activity for neuronal cell death from artery occlusion than CKJ291, although CKJ291 had a better probiotic activity. However, CKJ1 had much higher γ-PGA production than CKJ291, and γ-PGA contents might play a critical role in preventing ischemic stroke. These results suggest that the characteristics of *Bacillus* species and strains may play a critical role in the functionality of chungkookjang by modulating the γ-PGA contents and other metabolites of soybeans and the gut microbiota composition to elevate beneficial bacteria and SCFA production in a rodent ischemic stroke model by artery occlusion. The results of the present study cannot be directly applied to humans. Future human studies need to determine the preventive and alleviating effect of CKJ1 and CKJ291 on ischemic stroke.

## Figures and Tables

**Table 1:** The characteristics of two *Bacillus* spp. used for making chungkookjang, fermented soybeans.

	*B. amyloliquefaciens* SCGB 1	*B. subtilis* SCDB 291
Protease activity (diameter, cm)	2.3 ± 0.2	2.1 ± 0.1
Cellulase activity (diameter, cm)	1.2 ± 0.1	1.2 ± 0.1
Amylase activity (diameter, cm)	2.5 ± 0.2	2.1 ± 0.1 *
Thrombolytic activity (diameter, cm)	1.4 ± 0.0	1.6 ± 0.0 *
DPPH free removal activity (%)	15.0 ± 0.5	29.7 ± 1.2 *
Survival cells at 0.5% oxgall (%)	54.6 ± 1.1	72.9 ± 1.7 *
Survival cells at pH 2.0 (%)	52.1 ± 1.4	57.1 ± 1.2 *
Survival cells at 80°C (%)	55.7 ± 1.8	94.2 ± 1.6 *
Bile salt hydrolase activity (%)	0.0 ± 0.0	0.0 ± 0.0
CCD-18Co Cell adhesion (%)	62.1 ± 1.4	82.1 ± 0.9 *
γ-PGA (cm)	31 ± 0.9	22 ± 0.5 *

* Significantly different between two *Bacillus* spp. at *p* < 0.001.

**Table 2:** Energy metabolism.

	Control (*n* = 10)	Cooked Soybeans (*n* = 10)	CKJ1 (*n* = 10)	CKJ291 (*n* = 10)	Normal-Control (*n* = 10)
Body weight gain during 1–3 week period before artery occlusion (g)	2.87 ± 0.55 ^b^	3.05 ± 0.76 ^b^	7.27 ± 0.86 ^a^	7.16 ± 0.63 ^a^	2.57 ± 0.70 ^b^
Body weight gain during 1–3 week period after artery occlusion (g)	−3.20 ± 0.81 ^d^	1.52 ± 0.69 ^c^	5.82 ± 0.75 ^a^	3.04 ± 0.67 ^b^	2.71 ± 0.72 ^b^
Food intake during 1–3 week period before artery occlusion (g/day)	4.1 ± 0.51 ^b^	4.5 ± 0.53 ^b^	5.8 ± 0.50 ^a^	6.4 ± 0.57 ^a^	4.3 ± 0.55 ^b^
Food intake during 1–3 week period after artery occlusion (g/day)	4.1 ± 0.58	4.0 ± 0.48	3.9 ± 0.62	4.6 ± 0.63	4.1 ± 0.52
Isoflavonoid aglycones during 1–3 week period after artery occlusion (μg/day)	0 ± 0 ^d^	211 ± 24.2 ^c^	630 ± 99 ^b^	892 ± 122 ^a^	0 ± 0 ^d^
Isoflavonoid glycones during 1–3 week period after artery occlusion (μg/day)	0 ± 0 ^d^	1443 ± 173 ^a^	369 ± 59 ^b^	252 ± 34 ^c^	0 ± 0 ^d^

Values are means ± SD (*n* = 10). ^a,b,c,d^ values in the same row with different superscript letters were significantly different by Tukey’s test at *p* < 0.05.

**Table 3:** Proinflammatory cytokines in the blood and hippocampus on the 3rd week after artery occlusion.

	Control (*n* = 10)	Cooked Soybeans (*n* = 10)	CKJ1 (*n* = 10)	CKJ291 (*n* = 10)	Normal-Control (*n* = 10)
Serum IL-1β levels (pg/mL)	10.7 ± 1.17 ^a^	7.84 ± 0.88 ^b^	7.94 ± 0.84 ^b^	8.08 ± 0.91 ^b^	7.76 ± 0.79 ^b^
Serum TNF-α (pg/mL)	26.9 ± 2.15 ^a^	21.8 ± 2.05 ^b^	18.5 ± 2.03 ^c^	20.6 ± 1.98 ^bc^	17.9 ± 1.87 ^c^
Hippocampal lipid peroxides (MDA μmol/g tissue)	0.54 ± 0.08 ^a^	0.38 ± 0.05 ^b^	0.26 ± 0.05 ^c^	0.32 ± 0.04 ^b^	0.25 ± 0.05 ^c^
Relative mRNA expression of hippocampal TNF-α (AU)	2.2 ± 1.1 ^a^	1.0 ± 0.2 ^b^	0.98 ± 0.11 ^b^	1.1 ± 0.2 ^b^	1.0 ± 0.0 ^b^
Relative mRNA expression of hippocampal IL-1β (AU)	1.9 ± 0 ^a^	1.3 ± 0.2 ^b^	0.9 ± 0.2 ^c^	1.0 ± 0.2 ^c^	1.0 ± 0.0 ^c^
Relative mRNA expression of BDNF (AU)	0.54 ± 0.08 ^c^	0.81 ± 0.10 ^b^	0.93 ± 0.11 ^a^	0.86 ± 0.11 ^ab^	1.0 ± 0.0 ^a^

Values are means ± SD (*n* = 10). ^a,b,c^ values in the same row with different superscript letters were significantly different by Tukey’s test at *p* < 0.05. AU, arbitrary unit; MDA, malondialdehyde; TNF-α, tumor necrosis factor-α; IL-1β, interleukin-1β; BDNF, brain-derived neurotrophic factor.

**Table 4:** Blood flow and lipid profiles on the 3rd week after artery occlusion.

	Control (*n* = 10)	Cooked Soybeans (*n* = 10)	CKJ1 (*n* = 10)	CKJ291 (*n* = 10)	Normal-Control (*n* = 10)
Peak blood perfusion unit (BPU)	11.0 ± 3.94 ^d^	18.7 ± 3.73 ^c^	24.6 ± 4.01 ^b^	33.1 ± 4.89 ^a^	35.8 ± 4.44 ^a^
Periods to remove the clog (min)	3.23 ± 0.59 ^a^	2.51 ± 0.37 ^b^	1.51 ± 0.40 ^c^	1.42 ± 0.35 ^c^	1.54 ± 0.36 ^c^
Total cholesterol	181.2 ± 22.5 ^a^	149.1 ± 17.4 ^a^	151.4 ± 19.2 ^a^	146.2 ± 19.4 ^b^	139.4 ± 16.2 ^c^
HDL	33.5 ± 3.89 ^c^	33.6 ± 4.26 ^c^	50.1 ± 5.59 ^a^	47.7 ± 4.75 ^a^	42.2 ± 4.24 ^b^
LDL	105.5 ± 12.1 ^a^	92.0 ± 9.91 ^b^	71.6 ± 8.41 ^c^	69.7 ± 7.38 ^c^	69.4 ± 7.55 ^c^
Triglyceride	211.3 ± 22.0 ^a^	117.8 ± 9.39 ^c^	148.4 ± 16.8 ^b^	144.2 ± 12.3 ^b^	138.7 ± 14.2 ^b^
Fasting serum glucose levels (mg/dL)	111.1 ± 3.92 ^a^	94.7 ± 5.18 ^c^	92.2 ± 1.71 ^c^	102.2 ± 5.31 ^b^	91.6 ± 3.78 ^c^
Serum insulin levels (ng/mL)	0.52 ± 0.06 ^c^	0.60 ± 0.08 ^b^	0.65 ± 0.05 ^ab^	0.60 ± 0.08 ^b^	0.68 ± 0.05 ^a^
Fasting serum glucose at the 3rd week before artery occlusion (mg/dL)	95.4 ± 2.15 ^a^	89.5 ± 1.95 ^c^	88.8 ± 1.67 ^c^	92.2 ± 2.03 ^b^	95.5 ± 1.94 ^a^

Values are means ± SD (*n* = 10). ^a,b,c,d^ values in the same row with different superscript letters were significantly different by Tukey’s test at *p* < 0.05.

**Table 5:** Pancreatic β-cell mass.

	Control (*n* = 10)	Cooked Soybeans (*n* = 10)	CKJ1 (*n* = 10)	CKJ291 (*n* = 10)	Normal-Control (*n* = 10)
Individual β-cell size (μm^2^)	8.09 ± 0.92 ^a^	6.65 ± 0.73 ^b^	6.81 ± 0.71 ^b^	7.16 ± 0.74 ^ab^	6.24 ± 0.75 ^b^
β-cell area (%)	21.7 ± 2.76 ^c^	25.1 ± 3.02 ^b^	24.8 ± 2.95 ^b^	26.4 ± 3.04 ^a^	26.9 ± 3.12 ^a^
Total β-cell mass (mg)	0.98 ± 0.13 ^c^	1.24 ± 0.17 ^ab^	1.32 ± 0.19 ^a^	1.19 ± 0.12 ^b^	1.36 ± 0.15 ^a^
BrdU^+^ cells (% BrdU^+^ cells of islets)	5.03 ± 0.62 ^b^	5.83 ± 0.71 ^ab^	6.07 ± 0.69 ^a^	5.64 ± 0.65 ^ab^	5.96 ± 0.72 ^a^
Apoptosis (% apoptotic bodies of islets)	23.4 ± 2.61 ^a^	16.33 ± 1.94 ^c^	15.5 ± 1.82 ^c^	19.2 ± 2.25 ^b^	15.3 ± 1.67 ^c^

Values are means ± SD (*n* = 10). ^a,b,c^ values in the same row with different superscript letters were significantly different by Tukey’s test at *p* < 0.05.

**Table 6:** Serum short-chain fatty acid concentrations and fecal bacteria metabolism on the 21st day after artery occlusion.

	Control (*n* = 10)	Cooked Soybeans (*n* = 10)	CKJ1 (*n* = 10)	CKJ291 (*n* = 10)	Normal-Control (*n* = 10)
Serum acetate	0.89 ± 0.06	0.87 ± 0.07	0.94 ± 0.07	0.92 ± 0.09	0.88 ± 0.05
Serum propionate	0.27 ± 0.05	0.27 ± 0.03	0.27 ± 0.01	0.27 ± 0.02	0.26 ± 0.04
Serum butyrate	0.14 ± 0.02 ^b^	0.18 ± 0.02 ^a^	0.19 ± 0.02 ^a^	0.16 ± 0.02 ^ab^	0.16 ± 0.02 ^ab^
Chao	6897 ± 440 ^c^	9753 ± 574 ^a^	9259 ± 615 ^ab^	8957 ± 709 ^b^	9201 ± 657 ^ab^
Shannon index	4.8 ± 0.5 ^b^	5.6 ± 0.5 ^a^	5.5 ± 0.4 ^a^	5.6 ± 0.4 ^a^	5.6 ± 0.4 ^a^
Butanoate metabolism	1.08 ± 0.03 ^b^	1.13 ± 0.04 ^a^	1.19 ± 0.0.02 ^a^	1.10 ± 0.02 ^ab^	1.18 ± 0.04 ^a^
LPS biosynthesis	0.50 ± 0.07 ^a^	0.28 ± 0.02 ^b^	0.25 ± 0.06 ^b^	0.29 ± 0.05 ^b^	0.25 ± 0.03 ^b^
Starch and sucrose metabolism	1.94 ± 0.05 ^c^	2.35 ± 0.08 ^b^	2.80 ± 0.13 ^a^	2.67 ± 0.08 ^a^	2.32 ± 0.10 ^b^
Fatty acid metabolism	0.88 ± 0.03 ^a^	0.88 ± 0.03 ^a^	0.74 ± 0.03 ^b^	0.74 ± 0.01 ^b^	0.91 ± 0.04 ^a^
Branched-chain amino acid synthesis	0.85 ± 0.06 ^a^	0.73 ± 0.08 ^b^	0.53 ± 0.06 ^c^	0.67 ± 0.09 ^b^	0.71 ± 0.05 ^b^

Values are means ± SD (*n* = 10). ^a,b,c^ values in the same row with different superscript letters were significantly different by Tukey’s test at *p* < 0.05.

## Data Availability

The raw data involved in this study will be available by the authors to any qualified researcher.

## Supplementary Materials

The following are available online at https://www.mdpi.com/article/10.3390/foods10112697/s1, Table S1: Nutrient contents in soybeans and chungkookjang fermented with different *Bacillus* spp.
