# Supplementary material for: Alleviation of Neuronal Cell Death and Memory Deficit with Chungkookjang Made with Bacillus amyloliquefaciens and Bacillus subtilis Potentially through Promoting Gut–Brain Axis in Artery-Occluded Gerbils
Source: Foods. 2021 Nov 4;10(11):2697. doi: 10.3390/foods10112697 (PMC8619225; doi:10.3390/foods10112697)
Supplement: Supplementary file 1 [file foods-10-02697-s001.zip › foods-1407372-supplementary.pdf]

Table S1. Nutrient contents in soybeans and chungkookjang fermented with different *Bacillus* spp.

	Cooked soybeans	Chungkookjang SRCM 1	Chungkookjang SRCM 291
Carbohydrates (g/100g)	32.3	22.5	19.9
Proteins (g/100g)	15.9	18.2	19.6
Fats (g/100g)	30.3	36.1	36.6
Fiber (g/100g)	13.1	14.2	14.8
Ash (g/100g)	4.40	4.93	5.02
Genistein (μg/g)	13.2±0.20[c]	85.8±0.3[b]	104.0±5.4[a]
Daidzein (μg/g)	23.5±0.05[c]	66.4±1.6[b]	79.7±5.9[a]
Glycitein (μg/g)	16.1±0.38[a]	9.3±0.9[b]	10.2±1.9[b]
Isoflavonoid aglycones (μg/g)	52.8±0.7[c]	161.5±2.1[b]	193.9±4.6[a]
Genistin (μg/g)	204.6±20.0[a]	46.8±7.8[b]	29.4±6.3[c]
Daidzin (μg/g)	114.7±8.46[a]	40.3±2.3[b]	18.0±1.1[c]
Glycitin (μg/g)	41.5±0.81[a]	7.5±0.6[b]	7.4±0.9[b]
Isoflavonoid glycones (μg/g)	360.8±2.6[a]	94.6±3.8[b]	54.8±3.1[c]

Values are means ± SD (n=3). [a,b,c] values in the same row with different superscript letters were significantly different by Tukey’s test at $p<0.05$.
